# Understanding virtual primary healthcare with Indigenous populations: a rapid evidence review

**DOI:** 10.1186/s12913-023-09299-6

**Published:** 2023-03-29

**Authors:** Kayla M. Fitzpatrick, Meagan Ody, Danika Goveas, Stephanie Montesanti, Paige Campbell, Kathryn MacDonald, Lynden Crowshoe, Sandra Campbell, Pamela Roach

**Affiliations:** 1 School of Public Health, University of Alberta, Edmonton, AB Canada; 2 Department of Community Health Sciences, University of Calgary, Calgary, AB Canada; 3 Department of Family Medicine, University of Calgary, Calgary, AB Canada; 4 John W. Scott Health Sciences Library, University of Alberta, Edmonton, AB Canada

**Keywords:** Indigenous primary healthcare, Virtual care, Telehealth, Primary healthcare quality, Indigenous health

## Abstract

**Background:**

Virtual care has become an increasingly useful tool for the virtual delivery of care across the globe. With the unexpected emergence of COVID-19 and ongoing public health restrictions, it has become evident that the delivery of high-quality telemedicine is critical to ensuring the health and wellbeing of Indigenous peoples, especially those living in rural and remote communities.

**Methods:**

We conducted a rapid evidence review from August to December 2021 to understand how high quality Indigenous primary healthcare is defined in virtual modalities. After completing data extraction and quality appraisal, a total of 20 articles were selected for inclusion. The following question was used to guide the rapid review: *How is high quality Indigenous primary healthcare defined in virtual modalities?*

**Results:**

We discuss key limitations to the delivery of virtual care, including the increasing cost of technology, lack of accessibility, challenges with digital literacy, and language barriers. This review further yielded four main themes that highlight Indigenous virtual primary healthcare quality: (1) limitations and barriers of virtual primary healthcare, (2) Indigenous-centred virtual primary healthcare, (3) virtual Indigenous relationality, (4) collaborative approaches to ensuring holistic virtual care. Discussion: For virtual care to be Indigenous-centred, Indigenous leadership and users need to be partners in the development, implementation and evaluation of the intervention, service or program. In terms of virtual models of care, time must be allocated to educate Indigenous partners on digital literacy, virtual care infrastructure, benefits and limitations. Relationality and culture must be prioritized as well as digital health equity.

**Conclusion:**

These findings highlight important considerations for strengthening virtual primary healthcare approaches to meet the needs of Indigenous peoples worldwide.

**Supplementary Information:**

The online version contains supplementary material available at 10.1186/s12913-023-09299-6.

## Background

The delivery of quality primary healthcare (PHC) for Indigenous people and communities must be prioritized by local and national governments in Canada [1–3]. When accessing health services, Indigenous peoples experience inequities that stem from a lack of local and Indigenous-centred services, feelings of mistrust towards the healthcare system due to harmful past experiences, and jurisdictional and governmental disputes surrounding responsibility for Indigenous healthcare resources and delivery [4–7]. It’s well documented that Indigenous peoples face many barriers when trying to access PHC, such as the long-standing issue of a lack of PHC providers that provide care in Indigenous communities, which has been further exacerbated by the COVID-19 pandemic [8, 9]. Compounding this, PHC services are usually provided by non-Indigenous practitioners who follow western biomedical approaches, which ignore traditional healing practices and can oftentimes be incongruent with Indigenous ways of knowing [10, 11]. One potential solution to help improve access to and quality of Indigenous PHC in Indigenous communities is through virtual health care modalities. Virtual care is the provision of health-related services and information using telecommunications-based technologies. For this review, we will refer to the terminology “virtual care” to include telemedicine, telehealth, and other virtual modalities to provide PHC.

With the unexpected emergence of COVID-19, facilitation of virtual PHC has become more attainable and has the possibility to enhance the health and wellbeing of Indigenous peoples, especially those residing in rural and remote areas. Virtual care provides opportunities for specialty care (e.g., pediatricians) and for Indigenous PHC providers to be able to provide services in areas they may not have regular access to. However, there has been limited consultation with Indigenous communities in the development of these virtual care [12]. To ensure virtual care programs are aligned with community needs and acknowledge their specific cultural context, community engagement is an essential step in the creation of virtual PHC [12].

Indigenous-centred virtual care may offer a means to address existing healthcare gaps and enhance the health of Indigenous communities globally [12]. However, important to consider is the barriers Indigenous peoples face when accessing virtual care, including challenges with technology and lower broadband connectivity. Recent research highlights the consequences of inequitable access to virtual care, characterized as the ‘digital divide’ [13]. The digital divide is shaped by access and uptake of virtual PHC services and is often a contextual consideration for virtual care with Indigenous populations. Concerns surrounding the technological and cultural accessibility of virtual PHC services further highlight the need to explore virtual PHC [14] to ensure holistic aspects of health and self-management, health promotion and prevention are incorporated [2, 15, 16].

The rapid transition from in-person PHC service delivery to virtual modalities provides a critical opportunity to strengthen virtual care programs and services for Indigenous communities. The objective of this review is to synthesize the current evidence around virtual PHC services focused on Indigenous populations. This review examines the impacts and outcomes of virtual Indigenous PHC services, the barriers and enablers of successful Indigenous PHC virtual care, and existing virtual care frameworks. Moreover, environmental and contextual factors that impact Indigenous virtual PHC care are explored.

## Methods

Given the urgent need to understand Indigenous virtual PHC in the context of COVID-19, a rapid review methodology was purposefully chosen. Rapid reviews allow for a timely synthesis of available evidence on a particular topic and are commonly used for healthcare decision makers, knowledge users and policy [17, 18]. Rapid reviews include the development of a focused research question, a less developed search strategy, evidence searches, and more simplified data extraction and quality appraisal of the identified literature, when compared to traditional systematic reviews [18]. This rapid review was initially conducted from August to December 2021 and informed by rapid review methods outlined by the National Collaborating Centre for Methods and [19]. The following question was used to guide the rapid review: *How is high quality Indigenous PHC defined in virtual modalities?* A protocol has been registered and is published on the Open Science Framework Registries (10.17605/OSF.IO/VTUH7). This protocol was published after the search was conducted and reviewed by the expert librarian (SC). This is important to note, as we acknowledge we did not follow the JBI best practice guidelines on scoping reviews which recommends publishing the protocol prior to the study [20].

### Search strategy

The search strategy was developed to identify healthcare quality indicators, cultural safety indicators, and Indigenous perspectives of virtual care. A search was executed by an expert searcher/librarian (SC) on the following databases: OVID Medline, Ovid EMBASE, and EBSCO CINAHL using controlled vocabulary (eg: MeSH, Emtree, etc) and keywords representing the concepts “Indigenous people, “quality of care”, and “telehealth/remote care”. Searches were adjusted appropriately for different databases. Searches were conducted on August 10, 2021 and updated on January 23, 2023. All databases were searched from inception to present. Modified versions of search filters from the University of Alberta Health Sciences Search Filters were applied to retrieve some concepts (1–10). Results (602) were exported to the Covidence systematic review program, where duplicates (163) were removed. Detailed search strategies are available in Appendix 1.

### Selection criteria

**Fig. 1:**
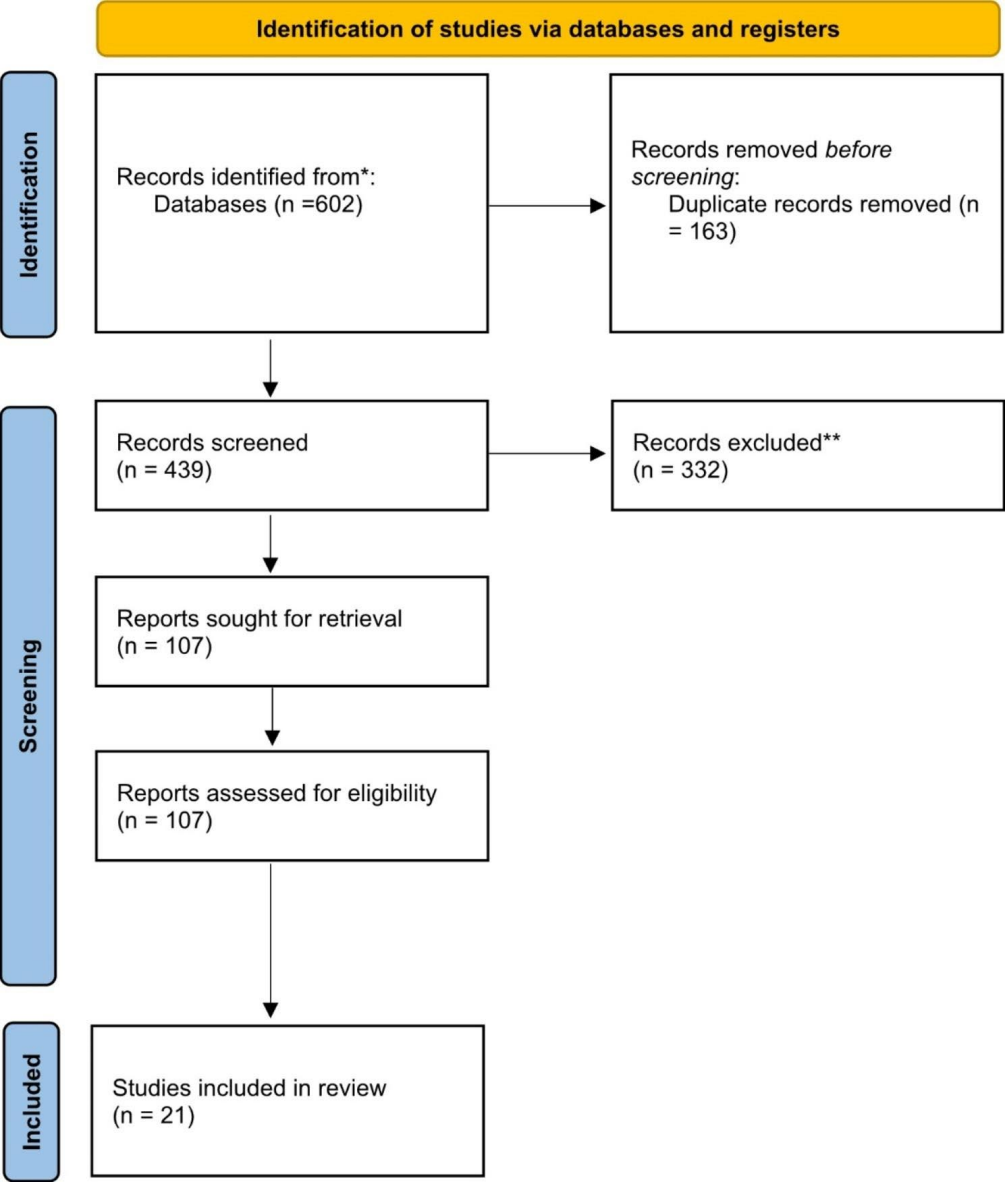
PRISMA Inclusion and Exclusion of Studies. (*Consider, if feasible to do so, reporting the number of records identified from each database or register searched (rather than the total number across all databases/registers). **If automation tools were used, indicate how many records were excluded by a human and how many were excluded by automation tools.)

The Population, Intervention, Comparator, Outcomes, and Design (PICOD) framework was employed to develop the eligibility criteria for this rapid review (Table 1). Publications were included in the review if they were (1) primary empirical studies (qualitative, quantitative, or mixed-methods), theoretical studies; reviews of empirical studies; implementation studies (2) focused on Indigenous peoples in Canada/USA/Australia/New Zealand (NZ) (3), focused on experiences of PHC for Indigenous populations in virtual modalities, and (4) interventions in virtual PHC delivery which included phone calls, text, video calls (e.g., Zoom, Facetime). Publications were excluded if they were (1) Indigenous populations outside of Canada/USA/Australia/NZ/circumpolar regions (2) thesis, commentaries, or opinion pieces (3) if the populations were non-Indigenous and (4) if the PHC modalities were not virtual interventions. Noteworthy, circumpolar regions was not included in our published protocol; however, circumpolar regions were included in our search strategy. Canada, USA, Australia, New Zealand and Circumpolar regions that are home to the Sami people in northern Europe have all experienced similar patterns of colonization and are currently facing very similar issues within the Indigenous populations [21, 22]. While each of these groups experience similar health issues with Indigenous populations, each healthcare system in each country differs in terms of privatized vs. public funding for healthcare. While this may be important context, it is important for us to note it was not something that we assessed or considered in this review. For title and abstract screening, each source was independently evaluated twice by authors (KM, MO, DG, PR). A full text review was conducted by authors (KM, MO, DG, KF, PC, PR) to verify which articles met inclusion criteria and any disagreements were resolved by discussion and with senior researchers (KF, PR) until a consensus was reached. See Fig. 1 for PRISMA [23] flowchart of publications included and excluded and appendix 2 for the PRISMA-S checklist.

**Table 1:** PICO(S) Statement

**Population**	Indigenous populations accessing PHC services in Canada/US/Australia/NZ/circumpolar region
**Intervention**	Interventions focused on experiences of PHC for Indigenous populations in online or telephone (virtual) modalities
**Comparison**	n/a
**Outcome**	Perspectives on high quality virtual PHC; Health care quality indicators; Cultural safety indicators
**Study design**	Primary empirical studies (qualitative, quantitative, or mixed methods), theoretical studies; reviews of empirical studies; implementation studies

### Data extraction

Each article was extracted twice by at least two different researchers (KF, DG, ML, PC, KM, PR) for consistency, and data was charted into a data extraction form in Microsoft Excel that included the source title, publication date, location, study characteristics, summary, and Critical Appraisal Skills Programme (CASP) quality appraisal (refer to Appendix 3 for the data extraction form). A summary of the data generated is available in Table 2 below and the full dataset generated from the extraction is available from the corresponding author on reasonable request.

### Data synthesis

Originally, as outlined in our protocol, we intended to use thematic content analysis, however, as we progressed in this review, we did not feel that quantifying words, themes or concepts as used in content [24] was the best method to examine Indigenous virtual care and instead felt that data reduction would produce more descriptive and representative results. We therefore, utilized Maxwell’ [25] and Miles and Huberman’s [26] qualitative thematic analysis technique of descriptive and pattern coding. Open-coding by authors (KF, MO, PC) was completed and then categorized to identify patterns, similarities, and differences throughout the data. Themes were reviewed and verified by an Indigenous health services researcher (PR), a public health and health policy researcher (SM), and a PHC service researcher and an Indigenous PHC provider (LC).

### Quality assessment

The quality of each study was evaluated using the CASP [27]. The CASP tool is designed as a pedagogic tool and there is no assigned score, if the answer is “yes” to the first 2/3 questions then the article can be considered of poor [27]. Quality assessments were divided in half and independently completed by 2 reviewers (MO, DG); any conflicts were resolved through consensus. No studies were appraised to be of poor quality; therefore, no articles were excluded from the review based on the CASP evaluation. Due to the urgent nature of this review, grey literature was not included.

## Results

In total, 21 studies met all criteria (Table 2) and included systematic reviews, RCT, qualitative studies, and case-control studies. Literature included work from Australia (7), Canada(7), New Zealand (5), USA (6) and circumpolar regions (2). Systematic reviews included in our review found that involving Indigenous communities in the design, implementation, and evaluation would benefit virtual care programs and mitigate costs of healthcare overall. Most of the data collection for the qualitative studies evaluated addressed the research issue but there were discrepancies in the focus on ‘satisfaction only’ surveys and the inability to extrapolate results further. Case studies exploring telehealth models for the treatment of specific health needs were found to be beneficial, however, it is important to keep in mind that many of these studies do not consider the social determinants of health leading to a narrower definition of health. Many studies insisted on ensuring Indigenous perspectives are utilized to provide better quality of the virtual program or service.

**Table 2:** Data from included studies

Author (publication year)	Title	Setting	Study Design	Sample	Main Purpose
Caffery, L. J. et al. (2018)	How telehealth facilitates the provision of culturally appropriate healthcare for Indigenous Australians	Australia	Qualitative Interviews	9 healthcare staff	To explore how telehealth facilitates or impedes the provision of culturally appropriate healthcare to Indigenous Australians.
Carswell, P. (2015)	Te Whiringa Ora: person-centred and integrated care in the Eastern Bay of Plenty, New Zealand	New Zealand	Case study: Participatory formative evaluation	53 patients; mix of Maori and New Zealand European	To understand how community-based programs can facilitate interdisciplinary care for patients and their families.
Fraser, S. et al. (2017)	Use of telehealth for health care of Indigenous peoples with chronic conditions: a systematic review	Australia, New Zealand, Canada, USA, Circumpolar regions	Systematic review	32 articles included	To explore the utility of telehealth for Indigenous peoples living with chronic health conditions.
Gibson, K. L. et al. (2011)	Conversations on telemental health: listening to remote and rural First Nations communities	Canada	Qualitative interviews	59 community members	To explore experiences with and perspectives of telemental health technologies from First Nations communities.
Ingemann, C. et al. (2020)	Patient experience studies in the circumpolar region: a scoping review	Circumpolar north	Scoping review	96 articles included for extraction	To investigate patient experiences within healthcare across the circumpolar north.
Jones, L. et al. (2017)	Development and Use of Health-Related Technologies in Indigenous Communities: Critical Review	Canada, Australia and USA	Critical review	34 articles included	To examine literature surrounding the use, adaptation, and development of assistive health technologies for older Indigenous adults.
Mashru, J. et al. (2017)	Management of Infectious diseases in remote northwestern Ontario with telemedicine videoconference consultations	Canada	Case study: Descriptive study	76 patients	To describe the implementation of a telemedicine-based infectious disease consultation service and patient satisfaction with the service.
Mendez, I. et al. (2013)	The use of remote presence for health care delivery in a northern Inuit community: a feasibility study	Canada	Case study and qualitative	Robot was activated 252 times in a 15 month period (exact sample size not provided)	To evaluate the feasibility of the RP-7 robot in improving the health of Inuit from a remote northern community.
Mooi, J. K. et al. (2012)	Teleoncology for Indigenous patients: The responses of patients and health workers	Australia	Case study: Descriptive study	9 Indigenous participants, 2 family members, 6 healthcare workers	To assess satisfaction with teleoncology and video consultations for Indigenous patients, their families, and health care workers.
Russell, S. et al. (2021)	Validation of the Kimberley Indigenous Cognitive Assessment short form (KICA screen) for telehealth	Australia	Prospective field trial	33 participants	To examine the utility of an Indigenous-specific dementia screening tool in a telehealth setting.
Volpe, T. et al. (2014)	Mental health services for Nunavut children and youth: evaluating a telepsychiatry pilot project	Canada	Pilot project	25 communities	To examine the utility of psychiatric consultation services using videoconferencing technology for health and mental health workers in Nunavut.
Sicotte, C. et al. (2011)	Use of telemedicine for haemodialysis in very remote areas: The Canadian first nations	Canada	Longitudinal study	19 individuals from 2 different communities	To compare the heath care utilization of patients receiving telehaemodialysis services between two communities.
Doorenbos, A. Z. et al. (2011)	Developing the Native People for Cancer Control Telehealth Network	USA	Case Study: Participatory formative evaluation	513 total patient encounters	To develop a telehealth network delivering postdiagnosis cancer care and education services for patients, families, and healthcare providers.
Smith, A. C. et al. (2012)	A mobile telemedicine enabled ear screening service for Indigenous children in Queensland: activity and outcomes in the first three years	Australia	Retrospective review	1053 children registered, 2111 screening assessments completed	To assess service activity and outcomes of a mobile telemedicine-enabled screening services.
Williams, M. et al. (2017)	Face-to-face versus telephone delivery of the Green Prescription for Maori and New Zealand Europeans with type-2 diabetes mellitus: influence on participation and health outcomes	New Zealand	Randomized Control Trial	138 patients; mix of Maori and New Zealand European	To compare the uptake and effectiveness of two different modes of delivery for the Green Prescription lifestyle program: face-to face vs. telephone-based services.
Caffery, L. J. et al. (2018)	Outcomes of using telehealth for the provision of healthcare to Aboriginal and Torres Strait Islander people: a systematic review	Australia	Systematic Review	14 articles included	To examine the reported outcomes of telehealth services delivered to Indigenous Australians.
Doorenbos, A. Z. et al. (2010)	Satisfaction With Telehealth for Cancer Support Groups in Rural American Indian and Alaska Native Communities	USA	Descriptive study	32 survey respondents	To assess information needs and satisfaction with telehealth support group services among cancer survivors in rural communities.
Kruse, C. S. et al. (2016)	Telemedicine Use in Rural Native American Communities in the Era of the ACA: a Systematic Literature Review	USA	Systematic review	15 articles included	To explore the cost, quality, and accessibility of telemedicine in rural Native American communities.
Potnek, M. F. (2020)	Urban American Indian Clinic Smoking Cessation Program	USA	Case-control study	5 program participants	To implement a nurse practitioner-led smoking cessation pilot program in an urban health centre.
Wikaire, E. et al. (2022)	Reducing healthcare inequities for Māori using Telehealth during COVID-19	New Zealand	Qualitative Interviews	5 Māori health professionals; 12 Māori patients	To investigate Māori experiences of telehealth consultations during the March 2020 COVID-19 lockdown.
Graham, F. et al. (2022)	Stakeholder perspectives of the sociotechnical requirements of a telehealth wheelchair assessment service in Aotearoa/New Zealand: A Qualitative Analysis	New Zealand	Qualitative Interviews	1 Māori health professional; 3 Māori wheelchair users.	To examine the design requirements of a telehealth wheelchair assessment service from the perspectives of key stakeholders such as wheelchair users and their families, including Indigenous (Māori) and health professionals.

From the included studies, four themes emerged on virtual delivery of Indigenous PHC: (1) limitations and barriers of virtual PHC, (2) Indigenous-centred virtual PHC, (3) virtual Indigenous relationality, (4) *Collaborative approaches to ensuring holistic virtual care*. To understand how to begin to define high quality Indigenous virtual PHC, we will first discuss the limitations and barriers to virtual care to understand what factors should be considered to produce high quality and what factors to avoid. We will then describe the components of high quality Indigenous virtual care which include Indigenous-centred, relationality, collaboration, and holistic care.

### Theme 1: Limitations and barriers of virtual primary healthcare

While virtual modalities are a promising solution to enable improved access to healthcare for Indigenous communities, there are several limitations and barriers that the authors highlighted for consideration. This included components such as challenges with a lack of face-to-face consultation, in addition to several cultural, technological, and educational barriers. With the inability to perform physical exams in the virtual space, one key issue identified was safety and whether or not a medical evaluation could be appropriately performed through a virtual [28–30]. In addition, the virtual setting limits opportunities to form trusting relationships between patient and [31, 32]. This can be problematic because building trust and rapport through relationships and community engagement is essential to ensuring the success and the provision of culturally safe health services to Indigenous [32, 33]. Noteworthy, some literature speaks to the history of Indian hospitals and ongoing systemic racism, and the long track record of distrust, particularly in the Canadian healthcare system with Indigenous populations, making rapport building and finding ways to build confidence between patient and provider even more of a priority [34]. Fraser and team [31] emphasized that “*Indigenous people have the right to culturally safe care… this can be facilitated through respectful listening to and meaningful engagement with Indigenous peoples and communities…”* ([31], p.11) .

Few studies looked at the development of implementation frameworks for Indigenous virtual healthcare programs and services. Without clear guidelines on how to engage with Indigenous communities in the virtual space to appropriately and effectively provide care, studies identified that there is an increased risk of harm and/or undue stress for patients [16, 35, 36] One study spoke to the lack of regional and national strategies and standards for the implementation of [37]. Adding to this, several studies pointed to the lack of cultural inclusion into frameworks and virtual care [31, 38, 39]. Similarly, Caffery and colleagues [40] discovered that there is a lack of evidence surrounding evaluation and evaluation frameworks for the delivery of virtual healthcare to Indigenous Australians which was confirmed by other [36] who discussed similar concerns in Canada, USA, and Australia. Another critical consideration is privacy of patients’ data as well as the privacy of a patient’s environment or space [37, 41]. Ensuring that virtual platforms are compliant with privacy regulations is a major ongoing challenge highlighted by several studies [28, 42]. Another consideration around privacy is related to relationships and trust with a provider which has been argued to be eroded in the virtual care environment [26, 27, 38]. Moreover, when considering the privacy of a patient’s environment, addressing complex trauma in the virtual setting is more difficult. Overcrowding and housing is a common problem in some Indigenous communities and can be problematic for individuals who are in particularly challenging living conditions to find a private location in their [43, 44].

Barriers associated with technology were noted often in the included articles. Many Indigenous communities experience lower socioeconomic status, may not have access to technology platforms and are commonly located in geographically rural areas with varying levels of bandwidth and internet [30–32, 45, 46]. As highlighted in the literature, the technology requires expensive equipment and training that is needed upfront [37]. Technology also requires sustainable long-term funding to be maintained, which is a common challenge within Indigenous communities and with virtual care programs that are being delivered from short-term research grant [32, 33, 37, 41]. In addition to technology, internet access, and infrastructure barriers, ‘digital literacy’ or the ‘digital divide’ which is a gap in access to digital technologies and infrastructure were cited as a major barrier which may be greater in at-risk [29, 41].

Other evident barriers to virtual care modalities included the time and expertise required to train healthcare staff about how virtual care technology works and to explain virtual care procedures to [31]. Due to the digital divide, telemedicine education and training are required for both providers and patients [46]. In addition, challenges were noted for virtual care providers in regards to adjusting to new procedures and practices in the day-to-day workflow [35]. A few studies found that the promotion of virtual care programs or knowledge of programs in community was also limited, again highlighting the importance of community engagement to increase awareness and buy-in from community [16, 46]. Further, lack of integration of traditional languages in virtual care technologies created barriers to access, which were cited in one article [31]. Lastly, virtual technologies are not accessible for all patients, such as those with medical disabilities (e.g., hearing loss, vision loss, dementia) [47]. The intersection of race, class, and health status all contribute to challenges experienced when implementing Indigenous virtual PHC which must be considered when designing programs of this nature and future research will be needed to better understand these intersections. While many barriers were identified, researchers described promising ways to mitigate some of these barriers and enhance virtual PHC for Indigenous populations.

### Theme 2: Indigenous-centred virtual primary healthcare

The majority of the articles included in this review were identified as being Indigenous-centred, meaning the program was developed with an Indigenous focus, while only one was Indigenous-led, meaning Indigenous communities and/or leaders led the design and implementation of the intervention. Consequently, all virtual care research and programs reviewed were not developed by and led by Indigenous communities (e.g., health centres), but rather developed in partnership with Indigenous communities and/or leadership. Several sources shared that the key components to successful Indigenous-centred virtual care implementation were engagement, community support, and partnership development, which in some cases, included training of local Indigenous [33, 38]. Indigenous-centred virtual PHC help to mitigate the barriers that were highlighted above such as trust.

A few studies highlighted the inclusion of Indigenous healthcare staff to support virtual care programs. The inclusion of Indigenous staff ensured Indigenous voices and values were a core component in the development and implementation of the virtual care [32, 39]. For example, one study described the positive impact of having a traditional healer present during the virtual care [40]. Another study discussed the grounding of their program in holistic and traditional principles (Whānau Ora) of the local Indigenous [33]. A piece of literature also supported investment in cultural competence with the additional inclusion of a trauma-informed lens as a way to ensure the virtual care programs were appropriate for Indigenous-centered care [33].

Several of the studies were developed through partnerships with governmental health bodies (e.g., Alberta Health Services) and Indigenous leadership in communities and/or organizations [36, 38, 39, 41]. One example described how researchers spent a considerable amount of time over several years, and continue to engage with local partners in all stages of implementation and [38]. It was evident that the studies with strong Indigenous partnerships also had a greater emphasis on culture in their virtual programs and [36, 38]. For example, one review described how a group in the USA prioritized meaningful engagement with partners and community, which resulted in the invention of the term “tele-spirituality” [36]. Tele-spirituality “describes consultations related to traditional medicine or ceremonial practices” ([36], p. 5). When virtual programs prioritize Indigenous voices, their uptake and overall sustainability are enhanced, as the community feels ownership over what they have [36]. On the contrary, the studies with less emphasis on Indigenous engagement or partnership were not as connected to respective Indigenous cultures, which could potentially signify a lack of cultural safety in the [39, 41]. Another example in the USA supporting American Indian health programs described how a telemedicine program did not include culture and that community was not consulted, and thereby, the lack of culture and Indigenous perspective was highlighted as a priority area for future [39].

### Theme 3: Virtual Indigenous relationality

#### Building relationships and trust

Relationality is a core concept for Indigenous communities worldwide [28], with relationships being described as the ‘spiritual and cultural foundations of Indigenous peoples,’ [48]. With the delivery of PHC in a virtual space, the emphasis on relationality was emphasized in articles and needs to be prioritized as patients and providers are unable to interact face to [30]. As identified by Carswell [33] building trust is a crucial step to enhancing the relationality between Indigenous patients and their healthcare providers. Two articles described how taking time to build mutual trust and understanding with Indigenous patients was integral to promoting adherence to their virtual PHC [29, 49]. Another article described how Indigenous patients “need to trust the service is providing something valuable to the patients,” which should be done through continual relationship building with healthcare providers [33]. A key enabler to strengthening relationality is building capacity within community. One article shared how continuous community engagement in the development and implementation of virtual PHC services provided an opportunity to build critical skills for community [41]. However, none of the articles mentioned concrete plans for capacity building that would otherwise enable Indigenous communities to sustain the virtual care programs over time.

#### Enhancing digital access

Indigenous patients need to feel confident about the technology and its infrastructure to ensure ease and comfort in navigating virtual care services. As mentioned earlier, one review highlighted how studies have reported that Indigenous peoples have privacy and confidentiality concerns surrounding communication technologies, which causes discomfort in navigating telehealth [31]. To address these concerns and barriers, one article highlighted the importance of providers taking time to address worries and explain how patient information is being protected with Indigenous patients [45]. Improving digital literacy is another way to ensure the success of virtual care programs. As highlighted above in theme 1, the “digital divide” has resulted in communities lacking the necessary infrastructure (e.g. quality internet service, broadband, ample cell towers) to sustain telehealth [30, 36]. Kruse [32] and team underscored the importance of funding and resource allocation toward improving technological infrastructure and enhancing digital literacy within Indigenous communities to prevent sustainability barriers [32]. Otherwise, the utility of telehealth may prove to be inadequate and underutilized.

#### Improved continuity of PHC and medical specialist outreach

Many Indigenous peoples reside in geographically rural and remote areas, which poses barriers to accessing timely PHC services. Telehealth provides a crucial opportunity to improve PHC access and delivery for Indigenous peoples by improving continuity of care and by enhancing accessibility for Indigenous patients seeking specialized care services. Articles highlighted how virtual care clinics increased opportunities for PHC to connect Indigenous clientele with medical specialists, who would otherwise rarely conduct in-community visits [28]. Furthermore, for some communities, specialist appointments conducted via telehealth eliminated transportation costs that would have been incurred if patients needed to travel to larger urban centres to receive that specialist medical care in-[28]. Some articles highlighted that another benefit to virtual specialist care is that it provides a continuity of care, which enables patients to receive consistent care from their [16], rather than the limited interactions during those infrequent physician visits to community.

### Theme 4: Collaborative approaches to ensuring holistic virtual care

Holistic care goes beyond the physiological metrics and examines the foundational relationships between physiological, psychological, social, spiritual, and cultural [50]. This collaborative strategy for addressing health [42] is considered integral to promoting quality healthcare for Indigenous [12, 51]. Only half of the articles mentioned the holistic aspects of care [28, 32, 37–41, 45–47, 52, 53], which adds to the literature described by Purdie et al., [51] and Fraser et al., [31] exposing existing gaps in Indigenous healthcare research from a holistic perspective. One good example mentioned the importance of integrating virtual care into holistic frameworks and addressed varied cultural conceptualizations of health and wellness, but stated that these aspects were not the focus of the [16]. Another article specifically elaborated on the importance of creating or maintaining aspects of holistic care in a virtual care [33]. Several critical and scoping reviews described that holistic care is essential to the delivery of comprehensive [31, 35, 36]. Fraser and team [31] conducted a systematic review on telehealth for Indigenous peoples with chronic disease and emphasized a clearly defined contemporary Aboriginal model of holistic care by Helen [50]. This included cultural, spiritual, social, emotional and physical dimensions and is influenced by traditional and contemporary components described as “*the intersection of both the layers and dimensions which creates the interconnectedness for a whole of life approach to Aboriginal wellbeing”* ([50], p. 8).

Successful incorporation of holistic care was often related to receiving information in one’s language and/or having a good [35, 54–60], but also focussed on shifting the provision of healthcare from treating the individual to an interdependent [33, 36]. Community-based decision making, involving patients in assessment processes, improving overall patient health literacy within Indigenous [49], and developing technology that includes family and [42, 61], all while incorporating culture and tradition into [16] can support a shift from individual self-management to a whole of community [33], leading to more holistic and integrative care. Diversifying points of access to services leads to an increase in program uptake, can demonstrate the inherent value of the service, and may increase the likelihood of engaging multiple [54], which leads to better health [51]. Some literature highlighted that western and colonial approaches to providing healthcare often compartmentalize and separate interrelated aspects that influence health [31], including access to services, the treatment of illness, and the definition of health [36].

## Discussion

The objective of this review was to synthesize the current evidence around virtual PHC services focused on Indigenous populations to be able to understand how quality Indigenous PHC is defined in virtual modalities. Our results show that for Indigenous virtual PHC to be of high quality it must be designed, implemented and evaluated in ethical and culturally-safe ways. This is increasingly important as more services shift to virtual delivery modalities in response to the COVID-19 pandemic and beyond. Moreover, we highlighted that virtual care is not inherently more appropriate or safer for Indigenous people than in-person delivery and the risk remains that virtual care can replicate current harmful systems of oppression of Indigenous people in the health system [62–66]. It is therefore important to consider Indigenous-led and Indigenous-centred virtual services that enable healthcare services to be both culturally safe and trauma-informed, in order to provide high quality care to Indigenous clients. This is encouraging for new virtual models of care to be designed in such a way that is congruent with the Truth and Reconciliation Commission of Canada’s Health Related Call to [67] and the United Nations Declaration on the Rights of Indigenous People outlining the importance of self-determination as an Indigenous determinant of [68].

Telemedicine is a useful tool for virtual healthcare delivery beyond the current pandemic context, as individuals living in rural and remote areas or those needing alternative accommodations could benefit from the continuity of virtual [69]. The range of technology-driven health services varies from telephone or virtual [69–73], text [74–76], store and forward [77, 78], web-based interventions and supports to the use of a remote presence robotic technology (RPRT)[37, 69]. Regardless of the modality used, it is imperative that the quality of virtual care meets or exceeds standards of in-person care and includes cultural and contextual considerations to ensure its success with Indigenous [79].

The digital divide is shaped by access and uptake of virtual care services and is often a contextual consideration for virtual care with Indigenous populations. Concerns surrounding the technological and cultural accessibility of virtual care services further highlight the need to explore patient experiences with virtual PHC through key [14] to ensure a focus on incorporating holistic aspects of health and self-management, health promotion and [2, 15, 16]. Further research is needed to examine how digital exclusion is experienced by diverse population groups, and across intersecting factors of gender, sex, age, geography, disability, race, ethnicity and [80]. One could argue that using a telephone for a phone call versus a video call is easier to access and better understood. Video conferencing takes more infrastructure, education, and time to set up; however, video conferencing provides enhances opportunities for relationship and rapport building.

For an intervention, service, or program to be Indigenous-centred, Indigenous leadership and users need to be partners in the development, implementation and evaluation of the intervention, service or program. In terms of virtual interventions, time must be allocated to educate Indigenous partners on digital literacy, virtual care infrastructure, benefits, and limitations. Research shows that clear guidance and support with technological infrastructure for health facilities and staff needs to be considered to ensure the successful delivery of Indigenous virtual healthcare and sustainable [79]. This requires an understanding of how to provide culturally competent and culturally safe care while being aware of digital determinants of health. For many Indigenous populations, experiences and impacts of digital determinants of health will be inherently intertwined with ongoing processes and policies of colonialism as the primary driver of Indigenous health [67] and so structural change must also be driven at the policy and legislative levels.

The digital determinants of health relate to our findings as it includes concepts such as access to digital resources, digital health literacy, beliefs about the potential for digital health to be helpful or harmful, values and cultural norms for use of digital resources, and integration of digital resources into a community and health [81]. Crawford and Serhal [81] developed a Digital Health Equity Framework which underscores the intersection of the digital determinants of health and digital health equity and the importance of using an ecological perspective when approaching digital health [81]. Indigenous virtual PHC initiatives can ensure digital health equity by identifying and addressing the potential gaps and needs within the digital determinants of health which have been highlighted in this review. For example, an Indigenous patient’s digital health care access and quality are shaped by their environment; in Canada, overcrowded homes are a reality in Indigenous communities which can result in a lack of privacy for patients, moreover, due to poverty, many may not have access to virtual care solutions at all [12]. If these factors are not considered when developing Indigenous virtual care interventions, quality of care will be negatively impacted, and digital health equity will therefore not be achieved. Worthy of mention here is a promising endeavour by the World Health Organization in the development of a strategy on digital health which will aim to “develop the infrastructure for information and communication technologies for health…[and] to promote equitable, affordable and universal access to their benefits” [80, 82]. One other positive step towards closing the digital divide was taken recently in Canada with the introduction of the Universal Broadband Fund, introduced by the Canadian Federal government in 2020. This was a C$1.75 billion investment to bring high-speed internet to rural and remote communities. As part of this initiative, up to C$50 million has been made available to support mobile internet projects that benefit Indigenous peoples in [80].

In addition to considering the digital determinants of health and digital health equity, a holistic approach to Indigenous virtual healthcare must be taken into account. This requires virtual care initiatives to also factor in relationality, spirituality, and self-determination. Further work needs to be done and directed by Indigenous people to understand how to best incorporate holistic approaches in a virtual environment. Virtual care training, digital literacy and cultural competence are often lacking in healthcare provider training. For healthcare workers who are expected to provide virtual healthcare to Indigenous populations, education about relationality, cultural humility, digital determinants of health and digital health equity should be incorporated into training as virtual healthcare interacts with economic, social, and cultural realities as well as with the social determinants of health. Moreover, attention must be paid to innovative ways to build trust and relationships with patients in a virtual space. One body of research has considered what is called “web-side” manner where healthcare providers are encouraged to ensure things such as their badge being visible, having the camera at eye level, and removing any background visual and audio [83]. Future studies should be conducted with a focus on culturally rooted perceptions of surveillance technologies used to support Indigenous patients, as this technology has the potential to replicate cycles of oppression and colonization leading to substantial barriers to virtual care. Co-design of virtual Indigenous PHC can help to mitigate these cycles and to be able to provide culturally safe and rooted care. Butler and her team described in 2022 that relevant co-design with First Nations Australians includes (1) First Nations Australians leadership (2) culturally grounded approach (3) respect (4) a benefit to First Nations communities (5) inclusive partnerships and (6) evidence-based decision making [84].

This is the first evidence review to the best of our knowledge that maps out the literature pertaining to Indigenous virtual PHC. Our results were limited to English language papers due to time and resource constraints. We do believe our search strategy was robust but as this was a rapid review the search was not comprehensive and did not provide quantitative measures of program effectiveness. As there is a steady shift to Indigenous virtual PHC modalities, it could be useful for scholars to continue this work to understand how Indigenous virtual PHC evolves. Future work could utilize a realist scoping review approach to provide information on how Indigenous virtual PHC is implemented and under what circumstances is it effective. Future work may also need to include translators to be more broadly inclusive of Indigenous experiences of virtual care beyond the regions included in this review and could provide valuable learnings from global Indigenous populations.

## Conclusion

The use of virtual healthcare technology is a promising innovative solution to providing more equitable PHC for Indigenous populations. Indigenous virtual PHC must consider technology and infrastructure barriers, access, digital health literacy skills, and other factors that can impact engagement with virtual care modalities. This means looking beyond individual factors to the health system as a whole to reduce virtual healthcare disparities for Indigenous peoples. Relationality and culture must be prioritized as well as digital health equity. Future research must ensure an understanding and inclusion of Indigenous-centered virtual PHC and the key domains of Indigenous health must be grounded in Indigenous values.

## Electronic supplementary material

Below is the link to the electronic supplementary material.

Supplementary Material 1

Supplementary Material 2

Supplementary Material 3

## Data Availability

Not applicable.
