# Supplementary material for: Understanding virtual primary healthcare with Indigenous populations: a rapid evidence review
Source: BMC Health Serv Res. 2023 Mar 29;23:303. doi: 10.1186/s12913-023-09299-6 (PMC10054202; doi:10.1186/s12913-023-09299-6)
Supplement: Supplementary file 1 — Supplementary Material 1 [file 12913_2023_9299_MOESM1_ESM.docx]

**Appendix 1**

**Ovid MEDLINE(R) ALL <1946 to August 09, 2021> <updated search to January 23, 2023>**

| # | Search Statement | Results |
| --- | --- | --- |
| 1 | exp Indigenous Canadians/ | 4130 |
| 2 | exp Health Services, Indigenous/ | 4009 |
| 3 | (exp Indians, North American/ or exp Inuits/ or exp Health Services, Indigenous/ or exp Ethnopharmacology/ or Athapaskan.mp. or Saulteaux.mp. or Wakashan.mp. or Cree.mp. or Dene.mp. or Inuit.mp. or Inuk.mp. or Inuvialuit*.mp. or Haida.mp. or Ktunaxa.mp. or Tsimshian.mp. or Gitsxan.mp. or Nisga'[a.mp](http://a.mp/). or Haisla.mp. or Heiltsuk.mp. or Oweenkeno.mp. or Kwakwaka'[wakw.mp](http://wakw.mp/). or Nuu chah [nulth.mp](http://nulth.mp/). or Tsilhqot'[in.mp](http://in.mp/). or Dakelh.mp. or Wet'suwet'[en.mp](http://en.mp/). or Sekani.mp. or Dunne-za.mp. or Dene.mp. or Tahltan.mp. or Kaska.mp. or Tagish.mp. or Tutchone.mp. or Nuxalk.mp. or Salish.mp. or Stl'[atlimc.mp](http://atlimc.mp/). or Nlaka'[pamux.mp](http://pamux.mp/). or Okanagan.mp. or Sec [wepmc.mp](http://wepmc.mp/). or Tlingit.mp. or Anishinaabe.mp. or Blackfoot.mp. or Nakoda.mp. or Tasttine.mp. or Tsuu T'[inia.mp](http://inia.mp/). or Gwich'[in.mp](http://in.mp/). or Han.mp. or Tagish.mp. or Tutchone.mp. or Algonquin.mp. or Nipissing.mp. or Ojibwa.mp. or Potawatomi.mp. or Innu.mp. or Maliseet.mp. or Mi'[kmaq.mp](http://kmaq.mp/). or Micmac.mp. or Passamaquoddy.mp. or Haudenosaunee.mp. or Cayuga.mp. or Mohawk.mp. or Oneida.mp. or Onodaga.mp. or Seneca.mp. or Tuscarora.mp. or Wyandot.mp. or Aboriginal*.mp. or Indigenous*.mp. or Metis.mp. or red [road.mp](http://road.mp/). or "on reserve".mp. or [off-reserve.mp](http://off-reserve.mp/). or First Nation.mp. or First Nations.mp. or Amerindian.mp. or (urban adj3 (Indian* or Native* or Aboriginal*)).mp. or [ethnomedicine.mp](http://ethnomedicine.mp/). or country food*.mp. or residential school*.mp. or ((exp Medicine, Traditional/ or traditional medicine*.mp.) not Chinese.mp.) or exp Shamanism/ or shaman*.mp. or traditional heal*.mp. or traditional food*.mp. or medicine [man.mp](http://man.mp/). or medicine [woman.mp](http://woman.mp/). or autochtone*.mp. or (Native* adj1 (man or men or women or woman or boy* or girl* or adolescent* or youth or youths or person* or adult or people* or Indian* or Nation or tribe* or tribal or band or bands)).mp.) and (exp Canada/ or (Canad* or British Columbia or Columbie Britannique or Alberta or Saskatchewan or Manitoba or Ontario or Quebec or Nova Scotia or New Brunswick or Newfoundland or Labrador or Prince Edward Island or Yukon Territory or NWT or Northwest Territories or Nunavut or Nunavik or Nunatsiavut or NunatuKavut).mp.) | 8757 |
| 4 | ((exp australia/ or (australia* or northern territory or tasmania or new south wales or Victoria or queensland).ti,ab.) and (exp oceanic ancestry group/ or aborigin*.ti,ab. or indigenous.ti,ab.)) or torres strait islander*.ti,ab. | 12223 |
| 5 | (maori or "tangata whenua").mp. or (New Zealand.mp. and (exp oceanic ancestry group/ or aborigin*.ti,ab. or indigenous.ti,ab.)) [mp=title, abstract, original title, name of substance word, subject heading word, floating sub-heading word, keyword heading word, organism supplementary concept word, protocol supplementary concept word, rare disease supplementary concept word, unique identifier, synonyms] | 5424 |
| 6 | ("A' ani" or Absaroka or Haaninin or Atsina or "Gros Ventre" or Acopsel or Tlacopsel or Lacopsel or Ahtna or Ahtena or Akenitsi or Occaneechi or Akokisa or Horcoquisa or Orcoquizas or Aleut or Unangax or Unangan or Alibamu or "Alabama Alsea" or Alutiiq or Sugpiag or "Pacific Yupik" or Amahami or Awaxawi or Androscoggin or Arosaguntacook or Ameriscoggin or Anishinaabeg or Chippewa or Anihsinape or Saulteaux or Apalachee or Aranama or "Texan Coahuilteca" or Tamique or Arikara or Sahnish or Arickaree or Adakadaho or Assiniboine or Hohe or Nakota or Nakoda or Nakona or "Atsa' Kudok-wa" or Awatixa or Bannock or "Snake Indian*" or Bidai or Quasmigdo or Biloxi or Blackfoot or Niitsitapi or Sikasikaitsitapi or Cahto or Kaipomo or Cahuilla or Ivilyuqaletem or Ivilyuat or Catawba or Inna or Iswa or Chemehuevi or Chickasaw or "Chilula Chimakum" or Aqokulo or Chimariko or Chiricahua or Tsokanende or Chitimacha or Chetimachan or Sitimacha or Chowanoke or Roanoke or Chumash or Ciboney or "Taino Ciwat" or Clatsop or Coos or Coosa or Uchis or Chiaha or Coste or Talisi or Coquille or Kokwell or Coso or Cowlitz or Taitnapam or "Crow Nation" or "Cui Ui Ticutta" or Cupeno or Kuupangaxwichem or Cupa or "Cup' ig" or Nunivak or "Dakota Oyate" or Lakota or Nakota or Santee or Teton or Sioux or Deadose or "Deg Xina" or "Deg Xit' an" or Kaiyuhkhotana or "Deg Hit' an" or "Dena' ina" or Tanaina or "Dichinanek' Hwt' ana" or "Upper Kuskokwim Athabascan*" or Kolchan or Goltsan or "Tundra Kolosh" or "Do lkabya" or Duwamish or Esselen or Eyak or "Gidi' tikadi" or Guwevkabaya or "Gwich' in" or Kutchin or Haida or Xaadas or Xaat or Halchidhoma or Havasupai or "Green Water People" or Hiratsa or Hiraaca or "Ho-chaaqa" or Winnebago or Holikachuk or Innoko or "Tlegon-khotana" or Hopi or "Houma-Louisiana" or Huaco or Waco or Hualapai or Hupa or Natinixwe or "Natinook-wa" or "Hwech' in" or Hankutchin or "Iroquois Confederacy" or "Hodinoso ni" or "Illinois Confedera*" or Ilinoweg or Illini or Inupiat or Inuit or Ioway or Baxoje or Jicarilla or Juaneno or Acjachemen or Jumano or Kalapuya or Clackama or Kalispel or "Pend d' Oreilles" or Qlispe or Karuk or Karok or "Chum-ne" or Katkoc or Kansa or Kanza or Kawaiisu or Nuwa or Kennebec or "Kinipekw Kittitas" or Klickitat or "Qwu' lh-hwai-pum" or "Awi-adshi" or Mahane or Wahnookt or "Koa' aga' itoka" or Keresan or Kichai or Kitsai or Keechi or "K' itaish" or Kiowa or Gaigwu or Cauigu or Kutjau or "Kwu-da" or "Tep-da" or Kitanemuk or Kittitas or Klickitat or "Qwu' lh-hwai-pum" or "Awi-adshi" or Mahane or Wahnookt or "Koa' aga' itoka" or Konkow or "Koop Ticutta" or Koyukon or Ktunaxa or Kootenai or Flathead or Kucadikadi or "Kotsa' va" or Kumeyaay or "Tipai-Ipai" or Kamia or Diegueno or Kwapa or Cocopah or Cucapa or "Xawitt kwnchawaay" or Lassik or Lenape or "Leni-Lenape" or Lipan or Luiseno or Payomkawichum or Madqwadabaya or "Desert Yavapai" or Mahican or Mohicans or Makah or Makuhadokado or Maliseet or Wolistoqiag or Manahoac or Mahock or Meipontsky or Mandan or Mattole or "Bear River" or "Tul' bush" or "Ni' ekeni" or Meherrin or Menominee or Mackinac or Mescalero or Myaamiaki or Kickapoo or Twigtwee or Missouria or Miwok or Miwuk or Moadokado or Modoc or Mohave or "Aha Makhav" or Mohawk or "Kaneng' hega" or Molala or Molale or Molele or Nyyhmy or Moosonee or "Moose Cree" or Monsonis or Multnomah or Chinook or Nabedache or Nabaydacu or Wawadishe or Nabiltse or Dakubetede or "Nacho Nyak Dun" or Tutchone or Nacono or "Na' isha" or Nanticoke or Navajo or Ndee or Nial or Niimiipu or "Nez Perce" or Watapala or Watapahlute or Nisenan or Nisqually or Nomlaki or Noamlakee or "Central Wintun" or Nongatl or Nottoway or Cheroenhaka or "Northern Cheyenne" or Ohlone or Costanoan or Omaha or "O' odham" or Pima or Papago or Osage or Otoe or Otse or "Ozav Dika" or Palus or Passamaquoddy or Pestomuhkati or Patiri or Petaros or Pastia or Patwin or "Southern Wintun" or Panis or Skidi or Pedee or Penobscot or "Petun Piipaash" or "Kokmalik' op" or Piscatawa or Doeg or Conoy or "Pit River" or Pomo or Kashaya or Ponca or Ponka or Pottawatomi or Bodewadmik or Powhatan or Puyallup or Spuyalepabs or Quapaw or Ugahxpa or Quechan or Yuma or Kwtsaan or Quileute or Salinan or Saponi or Monacan or Sapon or "Eastern Blackfoot" or Christanna or Sawawatodo or Serrano or Taaqtam or "Maarenga' yam" or Yuhaviatam or Shasta or Chasta or Sasti or Shoshone or Siletz or Sinkine or Sinkyone or "Siuslaw Umpqua" or Skitswish or "Schitsu' umash" or Snohomish or Snuqualmi or Sokoki or Missiquoi or Stillaguamish or Stoluckwamish or Suquamish or Sutaio or Swinomish or Skagit or Syilx or Okanagan or Sotaae or "Taga Ticutta" or Takelma or Dagelma or Taltushtuntede or Galice or "Tanan Gwich' in" or Taos or Taovaya or Tataviam or Alliklik or Tawakoni or Tahuacano or Tenino or Thawikila or Hathawekela or "Fort Ancient" or Tigua or Tillamook or Nehalem or Timbisha or Panamint or Timpanogos or Tlingit or "Toi Ticutta" or Tolowa or "Talawa Dini' " or Tongva or Gabrieleno or Fernandeno or Tobikhar or Tonkawa or Ticanwatic or Tsikip or Appalousa or Opelousa or Tsitsistas or Tubatulabal or Tukabatchee or Tuscarora or Tomahittan or Kuskarawock or Tutelo or Tutero or Totteroy or Tutera or Yusan or Tututni or Umatilla or Umpqua or Waccamaw or Waxmaw or Wadatika or "Harney Valley Paiute" or Wailiki or Waluulapam or "Walla Walla" or Walpapi or Huipui or Wampanoag or Massasoit or Wanapum or Wappo or Washoe or Wichita or Willapa or Kwalhioqua or "Wi pukba" or "Verde Valley Yavapai" or Wintu or "Northern Wintun" or Wiyot or "Wee' at" or Weyet or Yakama or "Yamosopo Tuviwarai" or Yaqui or Yoeme or Yatasi or Yattasih or "Yavbe' " or "Yavapai" or "Ysleta del Sur" or Yojuane or Yokuts or Mariposa or Yuki or Yupighyt or Yup'ik or Yupik or Yurok or "Olekwo'l" or Zuni).mp. | 15336 |
| 7 | ((Applegate or Delaware or Iowa or Ishak or Kaw or Kato or Spokane or Miami or Arkansas or Tali or Tunica or Han or Pawnee or "Coeur D' Alene" or Piscataway or Ree or Tula) adj3 (reservation* or nation or people or peoples or population or man or men or woman or women or child* or youth* or elder or elders or communit* or tribe or tribes or tribal or Indian*)).mp. | 11366 |
| 8 | or/1-7 | 53384 |
| 9 | exp Patient Satisfaction/ or ("patient satisfaction" or "patient experience" or "health care experience" or ("patient perception" adj3 quality)).mp. | 129685 |
| 10 | (culturally appropriate or culturally safe).mp. | 5893 |
| 11 | Quality Indicators, Health Care/ or (quality adj3 (indicator* or definition*)).mp. | 17267 |
| 12 | 9 or 10 or 11 | 151314 |
| 13 | exp Telemedicine/ or (((cyberthera* or telecare or telecollaborat* or teleconsult* or teleconference* or teleeducat* or telediagnos* or telehealth or teleguide* or telediagnos* or telelearn* or telemed* or telementor* or telemonitor* or teleneurol* or teleopth* or telepediatric* or telepresence* or telerehab* or telerobotic* or telescreen* or teletherap* or teletransmi* or mhealth or "m heath" or ehealth* or "e health" or (cyber or digital or remote* or distance* or tele)) adj2 (care or collaborat* or consult* or conference* or educat* or diagnos* or health or guide* or diagnos* or learn* or med* or mentor* or monitor* or presence* or screen* or therap* or transmi*)) or ((cyber or digital or distance* or tele or remote* or sms or phone* or internet or "web based" or telephone* or texting or "mobile app*" or Instagram or Snapchat or Facetime or GMeet* or hangout* or Skype or Zoom or Web-ex or WebEx or Bluejeans or Facebook or e-mail* or email* or "e chat" or echat or "social media" or "text message*" or "answering machine*" or "voice mail*" or "video conferenc*" or "video link*" or "video chat*") adj3 (consult* or support* or diagnos* or "follow-up*" or "health" or doctor* or "primary care" or clinic or clinics or clinician* or nurs* or psycholog* or therap* or intervention* or delivery))).mp. | 128588 |
| 14 | 8 and 12 and 13 | 119 |

**Embase <1974 to 2021 August 09> <updated search to January 23, 2023>**

| # | Search Statement | Results |
| --- | --- | --- |
| 1 | Indigenous people/ | 9067 |
| 2 | exp indigenous health care/ | 1213 |
| 3 | (canadian aboriginal/ or first nation/ or exp Inuits/ or Athapaskan.mp. or Saulteaux.mp. or Wakashan.mp. or Cree.mp. or Dene.mp. or Inuit.mp. or Inuk.mp. or Inuvialuit*.mp. or Haida.mp. or Ktunaxa.mp. or Tsimshian.mp. or Gitsxan.mp. or Nisga'[a.mp](http://a.mp/). or Haisla.mp. or Heiltsuk.mp. or Oweenkeno.mp. or Kwakwaka'[wakw.mp](http://wakw.mp/). or Nuu chah [nulth.mp](http://nulth.mp/). or Tsilhqot'[in.mp](http://in.mp/). or Dakelh.mp. or Wet'suwet'[en.mp](http://en.mp/). or Sekani.mp. or Dunne-za.mp. or Dene.mp. or Tahltan.mp. or Kaska.mp. or Tagish.mp. or Tutchone.mp. or Nuxalk.mp. or Salish.mp. or Stl'[atlimc.mp](http://atlimc.mp/). or Nlaka'[pamux.mp](http://pamux.mp/). or Okanagan.mp. or Sec [wepmc.mp](http://wepmc.mp/). or Tlingit.mp. or Anishinaabe.mp. or Blackfoot.mp. or Nakoda.mp. or Tasttine.mp. or Tsuu T'[inia.mp](http://inia.mp/). or Gwich'[in.mp](http://in.mp/). or Han.mp. or Tagish.mp. or Tutchone.mp. or Algonquin.mp. or Nipissing.mp. or Ojibwa.mp. or Potawatomi.mp. or Innu.mp. or Maliseet.mp. or Mi'[kmaq.mp](http://kmaq.mp/). or Micmac.mp. or Passamaquoddy.mp. or Haudenosaunee.mp. or Cayuga.mp. or Mohawk.mp. or Oneida.mp. or Onodaga.mp. or Seneca.mp. or Tuscarora.mp. or Wyandot.mp. or Aboriginal*.mp. or Indigenous*.mp. or Metis.mp. or red [road.mp](http://road.mp/). or "on reserve".mp. or [off-reserve.mp](http://off-reserve.mp/). or First Nation.mp. or First Nations.mp. or Amerindian.mp. or (urban adj3 (Indian* or Native* or Aboriginal*)).mp. or [ethnomedicine.mp](http://ethnomedicine.mp/). or country food*.mp. or residential school*.mp. or ((exp Medicine, Traditional/ or traditional medicine*.mp.) not Chinese.mp.) or exp Shamanism/ or shaman*.mp. or traditional heal*.mp. or traditional food*.mp. or medicine [man.mp](http://man.mp/). or medicine [woman.mp](http://woman.mp/). or autochtone*.mp. or (Native* adj1 (man or men or women or woman or boy* or girl* or adolescent* or youth or youths or person* or adult or people* or Indian* or Nation or tribe* or tribal or band or bands)).mp.) and (exp Canada/ or (Canad* or British Columbia or Columbie Britannique or Alberta or Saskatchewan or Manitoba or Ontario or Quebec or Nova Scotia or New Brunswick or Newfoundland or Labrador or Prince Edward Island or Yukon Territory or NWT or Northwest Territories or Nunavut or Nunavik or Nunatsiavut or NunatuKavut).mp.) | 9358 |
| 4 | australian aboriginal/ or ((exp australia/ or (australia* or northern territory or tasmania or new south wales or Victoria or queensland).ti,ab.) and (exp oceanic ancestry group/ or aborigin*.ti,ab. or indigenous.ti,ab.)) or torres strait islander*.ti,ab. | 13919 |
| 5 | (maori or "tangata whenua").mp. or (New Zealand.mp. and (exp oceanic ancestry group/ or aborigin*.ti,ab. or indigenous.ti,ab.)) [mp=title, abstract, heading word, drug trade name, original title, device manufacturer, drug manufacturer, device trade name, keyword, floating subheading word, candidate term word] | 6947 |
| 6 | american indians/ or alaska natives/ or exp eskimo-aleut people/ or ("A' ani" or Absaroka or Haaninin or Atsina or "Gros Ventre" or Acopsel or Tlacopsel or Lacopsel or Ahtna or Ahtena or Akenitsi or Occaneechi or Akokisa or Horcoquisa or Orcoquizas or Aleut or Unangax or Unangan or Alibamu or "Alabama Alsea" or Alutiiq or Sugpiag or "Pacific Yupik" or Amahami or Awaxawi or Androscoggin or Arosaguntacook or Ameriscoggin or Anishinaabeg or Chippewa or Anihsinape or Saulteaux or Apalachee or Aranama or "Texan Coahuilteca" or Tamique or Arikara or Sahnish or Arickaree or Adakadaho or Assiniboine or Hohe or Nakota or Nakoda or Nakona or "Atsa' Kudok-wa" or Awatixa or Bannock or "Snake Indian*" or Bidai or Quasmigdo or Biloxi or Blackfoot or Niitsitapi or Sikasikaitsitapi or Cahto or Kaipomo or Cahuilla or Ivilyuqaletem or Ivilyuat or Catawba or Inna or Iswa or Chemehuevi or Chickasaw or "Chilula Chimakum" or Aqokulo or Chimariko or Chiricahua or Tsokanende or Chitimacha or Chetimachan or Sitimacha or Chowanoke or Roanoke or Chumash or Ciboney or "Taino Ciwat" or Clatsop or Coos or Coosa or Uchis or Chiaha or Coste or Talisi or Coquille or Kokwell or Coso or Cowlitz or Taitnapam or "Crow Nation" or "Cui Ui Ticutta" or Cupeno or Kuupangaxwichem or Cupa or "Cup' ig" or Nunivak or "Dakota Oyate" or Lakota or Nakota or Santee or Teton or Sioux or Deadose or "Deg Xina" or "Deg Xit' an" or Kaiyuhkhotana or "Deg Hit' an" or "Dena' ina" or Tanaina or "Dichinanek' Hwt' ana" or "Upper Kuskokwim Athabascan*" or Kolchan or Goltsan or "Tundra Kolosh" or "Do lkabya" or Duwamish or Esselen or Eyak or "Gidi' tikadi" or Guwevkabaya or "Gwich' in" or Kutchin or Haida or Xaadas or Xaat or Halchidhoma or Havasupai or "Green Water People" or Hiratsa or Hiraaca or "Ho-chaaqa" or Winnebago or Holikachuk or Innoko or "Tlegon-khotana" or Hopi or "Houma-Louisiana" or Huaco or Waco or Hualapai or Hupa or Natinixwe or "Natinook-wa" or "Hwech' in" or Hankutchin or "Iroquois Confederacy" or "Hodinoso ni" or "Illinois Confedera*" or Ilinoweg or Illini or Inupiat or Inuit or Ioway or Baxoje or Jicarilla or Juaneno or Acjachemen or Jumano or Kalapuya or Clackama or Kalispel or "Pend d' Oreilles" or Qlispe or Karuk or Karok or "Chum-ne" or Katkoc or Kansa or Kanza or Kawaiisu or Nuwa or Kennebec or "Kinipekw Kittitas" or Klickitat or "Qwu' lh-hwai-pum" or "Awi-adshi" or Mahane or Wahnookt or "Koa' aga' itoka" or Keresan or Kichai or Kitsai or Keechi or "K' itaish" or Kiowa or Gaigwu or Cauigu or Kutjau or "Kwu-da" or "Tep-da" or Kitanemuk or Kittitas or Klickitat or "Qwu' lh-hwai-pum" or "Awi-adshi" or Mahane or Wahnookt or "Koa' aga' itoka" or Konkow or "Koop Ticutta" or Koyukon or Ktunaxa or Kootenai or Flathead or Kucadikadi or "Kotsa' va" or Kumeyaay or "Tipai-Ipai" or Kamia or Diegueno or Kwapa or Cocopah or Cucapa or "Xawitt kwnchawaay" or Lassik or Lenape or "Leni-Lenape" or Lipan or Luiseno or Payomkawichum or Madqwadabaya or "Desert Yavapai" or Mahican or Mohicans or Makah or Makuhadokado or Maliseet or Wolistoqiag or Manahoac or Mahock or Meipontsky or Mandan or Mattole or "Bear River" or "Tul' bush" or "Ni' ekeni" or Meherrin or Menominee or Mackinac or Mescalero or Myaamiaki or Kickapoo or Twigtwee or Missouria or Miwok or Miwuk or Moadokado or Modoc or Mohave or "Aha Makhav" or Mohawk or "Kaneng' hega" or Molala or Molale or Molele or Nyyhmy or Moosonee or "Moose Cree" or Monsonis or Multnomah or Chinook or Nabedache or Nabaydacu or Wawadishe or Nabiltse or Dakubetede or "Nacho Nyak Dun" or Tutchone or Nacono or "Na' isha" or Nanticoke or Navajo or Ndee or Nial or Niimiipu or "Nez Perce" or Watapala or Watapahlute or Nisenan or Nisqually or Nomlaki or Noamlakee or "Central Wintun" or Nongatl or Nottoway or Cheroenhaka or "Northern Cheyenne" or Ohlone or Costanoan or Omaha or "O' odham" or Pima or Papago or Osage or Otoe or Otse or "Ozav Dika" or Palus or Passamaquoddy or Pestomuhkati or Patiri or Petaros or Pastia or Patwin or "Southern Wintun" or Panis or Skidi or Pedee or Penobscot or "Petun Piipaash" or "Kokmalik' op" or Piscatawa or Doeg or Conoy or "Pit River" or Pomo or Kashaya or Ponca or Ponka or Pottawatomi or Bodewadmik or Powhatan or Puyallup or Spuyalepabs or Quapaw or Ugahxpa or Quechan or Yuma or Kwtsaan or Quileute or Salinan or Saponi or Monacan or Sapon or "Eastern Blackfoot" or Christanna or Sawawatodo or Serrano or Taaqtam or "Maarenga' yam" or Yuhaviatam or Shasta or Chasta or Sasti or Shoshone or Siletz or Sinkine or Sinkyone or "Siuslaw Umpqua" or Skitswish or "Schitsu' umash" or Snohomish or Snuqualmi or Sokoki or Missiquoi or Stillaguamish or Stoluckwamish or Suquamish or Sutaio or Swinomish or Skagit or Syilx or Okanagan or Sotaae or "Taga Ticutta" or Takelma or Dagelma or Taltushtuntede or Galice or "Tanan Gwich' in" or Taos or Taovaya or Tataviam or Alliklik or Tawakoni or Tahuacano or Tenino or Thawikila or Hathawekela or "Fort Ancient" or Tigua or Tillamook or Nehalem or Timbisha or Panamint or Timpanogos or Tlingit or "Toi Ticutta" or Tolowa or "Talawa Dini' " or Tongva or Gabrieleno or Fernandeno or Tobikhar or Tonkawa or Ticanwatic or Tsikip or Appalousa or Opelousa or Tsitsistas or Tubatulabal or Tukabatchee or Tuscarora or Tomahittan or Kuskarawock or Tutelo or Tutero or Totteroy or Tutera or Yusan or Tututni or Umatilla or Umpqua or Waccamaw or Waxmaw or Wadatika or "Harney Valley Paiute" or Wailiki or Waluulapam or "Walla Walla" or Walpapi or Huipui or Wampanoag or Massasoit or Wanapum or Wappo or Washoe or Wichita or Willapa or Kwalhioqua or "Wi pukba" or "Verde Valley Yavapai" or Wintu or "Northern Wintun" or Wiyot or "Wee' at" or Weyet or Yakama or "Yamosopo Tuviwarai" or Yaqui or Yoeme or Yatasi or Yattasih or "Yavbe' " or "Yavapai" or "Ysleta del Sur" or Yojuane or Yokuts or Mariposa or Yuki or Yupighyt or Yup'ik or Yupik or Yurok or "Olekwo'l" or Zuni).mp. | 37287 |
| 7 | ((Applegate or Delaware or Iowa or Ishak or Kaw or Kato or Spokane or Miami or Arkansas or Tali or Tunica or Han or Pawnee or "Coeur D' Alene" or Piscataway or Ree or Tula) adj3 (reservation* or nation or people or peoples or population or man or men or woman or women or child* or youth* or elder or elders or communit* or tribe or tribes or tribal or Indian*)).mp. | 14012 |
| 8 | or/1-7 | 81050 |
| 9 | exp Patient Satisfaction/ or ("patient satisfaction" or "patient experience" or "health care experience" or ("patient perception" adj3 quality)).mp. | 187606 |
| 10 | (culturally appropriate or culturally safe).mp. | 7055 |
| 11 | health care quality/ or (quality adj3 (indicator* or definition*)).mp. | 265430 |
| 12 | 9 or 10 or 11 | 444238 |
| 13 | exp Telemedicine/ or (((cyberthera* or telecare or telecollaborat* or teleconsult* or teleconference* or teleeducat* or telediagnos* or telehealth or teleguide* or telediagnos* or telelearn* or telemed* or telementor* or telemonitor* or teleneurol* or teleopth* or telepediatric* or telepresence* or telerehab* or telerobotic* or telescreen* or teletherap* or teletransmi* or mhealth or "m heath" or ehealth* or "e health" or (cyber or digital or remote* or distance* or tele)) adj2 (care or collaborat* or consult* or conference* or educat* or diagnos* or health or guide* or diagnos* or learn* or med* or mentor* or monitor* or presence* or screen* or therap* or transmi*)) or ((cyber or digital or distance* or tele or remote* or sms or phone* or internet or "web based" or telephone* or texting or "mobile app*" or Instagram or Snapchat or Facetime or GMeet* or hangout* or Skype or Zoom or Web-ex or WebEx or Bluejeans or Facebook or e-mail* or email* or "e chat" or echat or "social media" or "text message*" or "answering machine*" or "voice mail*" or "video conferenc*" or "video link*" or "video chat*") adj3 (consult* or support* or diagnos* or "follow-up*" or "health" or doctor* or "primary care" or clinic or clinics or clinician* or nurs* or psycholog* or therap* or intervention* or delivery))).mp. | 170534 |
| 14 | 8 and 12 and 13 | 201 |

**CINAHL Plus with Full Text**

**Limiters/Expanders - Search modes - Find all my search terms**

**<updated search to January 23, 2023>**

| # | Query | Results |
| --- | --- | --- |
| S24 | s18 NOT s23 | 282 |
| S23 | S19 OR S20 OR S21 OR S22 | 1,446 |
| S22 | Waco, Texas | 24 |
| S21 | Wee-Functional Independence Measure | 6 |
| S20 | "kansas city" | 941 |
| S19 | (MH "Omaha System+") | 475 |
| S18 | S8 AND S14 AND S17 | 313 |
| S17 | S15 OR S16 | 81,819 |
| S16 | (((cyberthera* or telecare or telecollaborat* or teleconsult* or teleconference* or teleeducat* or telediagnos* or telehealth or teleguide* or telediagnos* or telelearn* or telemed* or telementor* or telemonitor* or teleneurol* or teleopth* or telepediatric* or telepresence* or telerehab* or telerobotic* or telescreen* or teletherap* or teletransmi* or mhealth or "m heath" or ehealth* or "e health" or (cyber or digital or remote* or distance* or tele)) N2 (care or collaborat* or consult* or conference* or educat* or diagnos* or health or guide* or diagnos* or learn* or med* or mentor* or monitor* or presence* or screen* or therap* or transmi*)) or ((cyber or digital or distance* or tele or remote* or sms or phone* or internet or "web based" or telephone* or texting or "mobile app*" or Instagram or Snapchat or Facetime or GMeet* or hangout* or Skype or Zoom or Web-ex or WebEx or Bluejeans or Facebook or e-mail* or email* or "e chat" or echat or "social media" or "text message*" or "answering machine*" or "voice mail*" or "video conferenc*" or "video link*" or "video chat*") N3 (consult* or support* or diagnos* or "follow-up*" or "health" or doctor* or "primary care" or clinic or clinics or clinician* or nurs* or psycholog* or therap* or intervention* or delivery))) | 60,727 |
| S15 | (MH "Telehealth+") OR (MH "Telemedicine+") OR (MH "Remote Consultation") OR (MH "Telerehabilitation") OR (MH "Telenursing") OR (MH "Telenutrition") OR (MH "Telepsychiatry") | 34,394 |
| S14 | S9 OR S10 OR S11 OR S12 OR S13 | 968,439 |
| S13 | (MH "Quality of Health Care+") | 914,191 |
| S12 | quality N3 (indicator* or definition*) | 8,711 |
| S11 | (cultural* N3 appropriate* or cultural* N3 safe*) | 6,586 |
| S10 | (MH "Patient Satisfaction+") OR (MH "Patient Preference") | 63,690 |
| S9 | ("patient satisfaction" or "patient experience" or "health care experience" or ("patient perception" N3 quality) | 73,953 |
| S8 | S1 or s2 or s3 or s4 or s5 or s6 or s7 | 37,110 |
| S7 | (((Applegate or Delaware or Iowa or Ishak or Kaw or Kato or Spokane or Miami or Arkansas or Tali or Tunica or Han or Pawnee or "Coeur D' Alene" or Piscataway or Ree or Tula) N3 (reservation* or nation or people or peoples or population or man or men or woman or women or child* or youth* or elder or elders or communit* or tribe or tribes or tribal or Indian*))) AND (S1 OR S2 OR S3 OR S4 OR S5 OR S6) | 2,689 |
| S6 | ((Applegate or Delaware or Iowa or Ishak or Kaw or Kato or Spokane or Miami or Arkansas or Tali or Tunica or Han or Pawnee or "Coeur D' Alene" or Piscataway or Ree or Tula) N3 (reservation* or nation or people or peoples or population or man or men or woman or women or child* or youth* or elder or elders or communit* or tribe or tribes or tribal or Indian*)) | 2,689 |
| S5 | TI ("A' ani" or Absaroka or Haaninin or Atsina or "Gros Ventre" or Acopsel or Tlacopsel or Lacopsel or Ahtna or Ahtena or Akenitsi or Occaneechi or Akokisa or Horcoquisa or Orcoquizas or Aleut or Unangax or Unangan or Alibamu or "Alabama Alsea" or Alutiiq or Sugpiag or "Pacific Yupik" or Amahami or Awaxawi or Androscoggin or Arosaguntacook or Ameriscoggin or Anishinaabeg or Chippewa or Anihsinape or Saulteaux or Apalachee or Aranama or "Texan Coahuilteca" or Tamique or Arikara or Sahnish or Arickaree or Adakadaho or Assiniboine or Hohe or Nakota or Nakoda or Nakona or "Atsa' Kudok-wa" or Awatixa or Bannock or "Snake Indian*" or Bidai or Quasmigdo or Biloxi or Blackfoot or Niitsitapi or Sikasikaitsitapi or Cahto or Kaipomo or Cahuilla or Ivilyuqaletem or Ivilyuat or Catawba or Inna or Iswa or Chemehuevi or Chickasaw or "Chilula Chimakum" or Aqokulo or Chimariko or Chiricahua or Tsokanende or Chitimacha or Chetimachan or Sitimacha or Chowanoke or Roanoke or Chumash or Ciboney or "Taino Ciwat" or Clatsop or Coos or Coosa or Uchis or Chiaha or Coste or Talisi or Coquille or Kokwell or Coso or Cowlitz or Taitnapam or "Crow Nation" or "Cui Ui Ticutta" or Cupeno or Kuupangaxwichem or Cupa or "Cup' ig" or Nunivak or "Dakota Oyate" or Lakota or Nakota or Santee or Teton or Sioux or Deadose or "Deg Xina" or "Deg Xit' an" or Kaiyuhkhotana or "Deg Hit' an" or "Dena' ina" or Tanaina or "Dichinanek' Hwt' ana" or "Upper Kuskokwim Athabascan*" or Kolchan or Goltsan or "Tundra Kolosh" or "Do lkabya" or Duwamish or Esselen or Eyak or "Gidi' tikadi" or Guwevkabaya or "Gwich' in" or Kutchin or Haida or Xaadas or Xaat or Halchidhoma or Havasupai or "Green Water People" or Hiratsa or Hiraaca or "Ho-chaaqa" or Winnebago or Holikachuk or Innoko or "Tlegon-khotana" or Hopi or "Houma-Louisiana" or Huaco or Waco or Hualapai or Hupa or Natinixwe or "Natinook-wa" or "Hwech' in" or Hankutchin or "Iroquois Confederacy" or "Hodinoso ni" or "Illinois Confedera*" or Ilinoweg or Illini or Inupiat or Inuit or Ioway or Baxoje or Jicarilla or Juaneno or Acjachemen or Jumano or Kalapuya or Clackama or Kalispel or "Pend d' Oreilles" or Qlispe or Karuk or Karok or "Chum-ne" or Katkoc or Kansa or Kanza or Kawaiisu or Nuwa or Kennebec or "Kinipekw Kittitas" or Klickitat or "Qwu' lh-hwai-pum" or "Awi-adshi" or Mahane or Wahnookt or "Koa' aga' itoka" or Keresan or Kichai or Kitsai or Keechi or "K' itaish" or Kiowa or Gaigwu or Cauigu or Kutjau or "Kwu-da" or "Tep-da" or Kitanemuk or Kittitas or Klickitat or "Qwu' lh-hwai-pum" or "Awi-adshi" or Mahane or Wahnookt or "Koa' aga' itoka" or Konkow or "Koop Ticutta" or Koyukon or Ktunaxa or Kootenai or Flathead or Kucadikadi or "Kotsa' va" or Kumeyaay or "Tipai-Ipai" or Kamia or Diegueno or Kwapa or Cocopah or Cucapa or "Xawitt kwnchawaay" or Lassik or Lenape or "Leni-Lenape" or Lipan or Luiseno or Payomkawichum or Madqwadabaya or "Desert Yavapai" or Mahican or Mohicans or Makah or Makuhadokado or Maliseet or Wolistoqiag or Manahoac or Mahock or Meipontsky or Mandan or Mattole or "Bear River" or "Tul' bush" or "Ni' ekeni" or Meherrin or Menominee or Mackinac or Mescalero or Myaamiaki or Kickapoo or Twigtwee or Missouria or Miwok or Miwuk or Moadokado or Modoc or Mohave or "Aha Makhav" or Mohawk or "Kaneng' hega" or Molala or Molale or Molele or Nyyhmy or Moosonee or "Moose Cree" or Monsonis or Multnomah or Chinook or Nabedache or Nabaydacu or Wawadishe or Nabiltse or Dakubetede or "Nacho Nyak Dun" or Tutchone or Nacono or "Na' isha" or Nanticoke or Navajo or Ndee or Nial or Niimiipu or "Nez Perce" or Watapala or Watapahlute or Nisenan or Nisqually or Nomlaki or Noamlakee or "Central Wintun" or Nongatl or Nottoway or Cheroenhaka or "Northern Cheyenne" or Ohlone or Costanoan or Omaha or "O' odham" or Pima or Papago or Osage or Otoe or Otse or "Ozav Dika" or Palus or Passamaquoddy or Pestomuhkati or Patiri or Petaros or Pastia or Patwin or "Southern Wintun" or Panis or Skidi or Pedee or Penobscot or "Petun Piipaash" or "Kokmalik' op" or Piscatawa or Doeg or Conoy or "Pit River" or Pomo or Kashaya or Ponca or Ponka or Pottawatomi or Bodewadmik or Powhatan or Puyallup or Spuyalepabs or Quapaw or Ugahxpa or Quechan or Yuma or Kwtsaan or Quileute or Salinan or Saponi or Monacan or Sapon or "Eastern Blackfoot" or Christanna or Sawawatodo or Serrano or Taaqtam or "Maarenga' yam" or Yuhaviatam or Shasta or Chasta or Sasti or Shoshone or Siletz or Sinkine or Sinkyone or "Siuslaw Umpqua" or Skitswish or "Schitsu' umash" or Snohomish or Snuqualmi or Sokoki or Missiquoi or Stillaguamish or Stoluckwamish or Suquamish or Sutaio or Swinomish or Skagit or Syilx or Okanagan or Sotaae or "Taga Ticutta" or Takelma or Dagelma or Taltushtuntede or Galice or "Tanan Gwich' in" or Taos or Taovaya or Tataviam or Alliklik or Tawakoni or Tahuacano or Tenino or Thawikila or Hathawekela or "Fort Ancient" or Tigua or Tillamook or Nehalem or Timbisha or Panamint or Timpanogos or Tlingit or "Toi Ticutta" or Tolowa or "Talawa Dini' " or Tongva or Gabrieleno or Fernandeno or Tobikhar or Tonkawa or Ticanwatic or Tsikip or Appalousa or Opelousa or Tsitsistas or Tubatulabal or Tukabatchee or Tuscarora or Tomahittan or Kuskarawock or Tutelo or Tutero or Totteroy or Tutera or Yusan or Tututni or Umatilla or Umpqua or Waccamaw or Waxmaw or Wadatika or "Harney Valley Paiute" or Wailiki or Waluulapam or "Walla Walla" or Walpapi or Huipui or Wampanoag or Massasoit or Wanapum or Wappo or Washoe or Wichita or Willapa or Kwalhioqua or "Wi pukba" or "Verde Valley Yavapai" or Wintu or "Northern Wintun" or Wiyot or "Wee' at" or Weyet or Yakama or "Yamosopo Tuviwarai" or Yaqui or Yoeme or Yatasi or Yattasih or "Yavbe' " or "Yavapai" or "Ysleta del Sur" or Yojuane or Yokuts or Mariposa or Yuki or Yupighyt or Yup'ik or Yupik or Yurok or "Olekwo'l" or Zuni) or AB ("A' ani" or Absaroka or Haaninin or Atsina or "Gros Ventre" or Acopsel or Tlacopsel or Lacopsel or Ahtna or Ahtena or Akenitsi or Occaneechi or Akokisa or Horcoquisa or Orcoquizas or Aleut or Unangax or Unangan or Alibamu or "Alabama Alsea" or Alutiiq or Sugpiag or "Pacific Yupik" or Amahami or Awaxawi or Androscoggin or Arosaguntacook or Ameriscoggin or Anishinaabeg or Chippewa or Anihsinape or Saulteaux or Apalachee or Aranama or "Texan Coahuilteca" or Tamique or Arikara or Sahnish or Arickaree or Adakadaho or Assiniboine or Hohe or Nakota or Nakoda or Nakona or "Atsa' Kudok-wa" or Awatixa or Bannock or "Snake Indian*" or Bidai or Quasmigdo or Biloxi or Blackfoot or Niitsitapi or Sikasikaitsitapi or Cahto or Kaipomo or Cahuilla or Ivilyuqaletem or Ivilyuat or Catawba or Inna or Iswa or Chemehuevi or Chickasaw or "Chilula Chimakum" or Aqokulo or Chimariko or Chiricahua or Tsokanende or Chitimacha or Chetimachan or Sitimacha or Chowanoke or Roanoke or Chumash or Ciboney or "Taino Ciwat" or Clatsop or Coos or Coosa or Uchis or Chiaha or Coste or Talisi or Coquille or Kokwell or Coso or Cowlitz or Taitnapam or "Crow Nation" or "Cui Ui Ticutta" or Cupeno or Kuupangaxwichem or Cupa or "Cup' ig" or Nunivak or "Dakota Oyate" or Lakota or Nakota or Santee or Teton or Sioux or Deadose or "Deg Xina" or "Deg Xit' an" or Kaiyuhkhotana or "Deg Hit' an" or "Dena' ina" or Tanaina or "Dichinanek' Hwt' ana" or "Upper Kuskokwim Athabascan*" or Kolchan or Goltsan or "Tundra Kolosh" or "Do lkabya" or Duwamish or Esselen or Eyak or "Gidi' tikadi" or Guwevkabaya or "Gwich' in" or Kutchin or Haida or Xaadas or Xaat or Halchidhoma or Havasupai or "Green Water People" or Hiratsa or Hiraaca or "Ho-chaaqa" or Winnebago or Holikachuk or Innoko or "Tlegon-khotana" or Hopi or "Houma-Louisiana" or Huaco or Waco or Hualapai or Hupa or Natinixwe or "Natinook-wa" or "Hwech' in" or Hankutchin or "Iroquois Confederacy" or "Hodinoso ni" or "Illinois Confedera*" or Ilinoweg or Illini or Inupiat or Inuit or Ioway or Baxoje or Jicarilla or Juaneno or Acjachemen or Jumano or Kalapuya or Clackama or Kalispel or "Pend d' Oreilles" or Qlispe or Karuk or Karok or "Chum-ne" or Katkoc or Kansa or Kanza or Kawaiisu or Nuwa or Kennebec or "Kinipekw Kittitas" or Klickitat or "Qwu' lh-hwai-pum" or "Awi-adshi" or Mahane or Wahnookt or "Koa' aga' itoka" or Keresan or Kichai or Kitsai or Keechi or "K' itaish" or Kiowa or Gaigwu or Cauigu or Kutjau or "Kwu-da" or "Tep-da" or Kitanemuk or Kittitas or Klickitat or "Qwu' lh-hwai-pum" or "Awi-adshi" or Mahane or Wahnookt or "Koa' aga' itoka" or Konkow or "Koop Ticutta" or Koyukon or Ktunaxa or Kootenai or Flathead or Kucadikadi or "Kotsa' va" or Kumeyaay or "Tipai-Ipai" or Kamia or Diegueno or Kwapa or Cocopah or Cucapa or "Xawitt kwnchawaay" or Lassik or Lenape or "Leni-Lenape" or Lipan or Luiseno or Payomkawichum or Madqwadabaya or "Desert Yavapai" or Mahican or Mohicans or Makah or Makuhadokado or Maliseet or Wolistoqiag or Manahoac or Mahock or Meipontsky or Mandan or Mattole or "Bear River" or "Tul' bush" or "Ni' ekeni" or Meherrin or Menominee or Mackinac or Mescalero or Myaamiaki or Kickapoo or Twigtwee or Missouria or Miwok or Miwuk or Moadokado or Modoc or Mohave or "Aha Makhav" or Mohawk or "Kaneng' hega" or Molala or Molale or Molele or Nyyhmy or Moosonee or "Moose Cree" or Monsonis or Multnomah or Chinook or Nabedache or Nabaydacu or Wawadishe or Nabiltse or Dakubetede or "Nacho Nyak Dun" or Tutchone or Nacono or "Na' isha" or Nanticoke or Navajo or Ndee or Nial or Niimiipu or "Nez Perce" or Watapala or Watapahlute or Nisenan or Nisqually or Nomlaki or Noamlakee or "Central Wintun" or Nongatl or Nottoway or Cheroenhaka or "Northern Cheyenne" or Ohlone or Costanoan or Omaha or "O' odham" or Pima or Papago or Osage or Otoe or Otse or "Ozav Dika" or Palus or Passamaquoddy or Pestomuhkati or Patiri or Petaros or Pastia or Patwin or "Southern Wintun" or Panis or Skidi or Pedee or Penobscot or "Petun Piipaash" or "Kokmalik' op" or Piscatawa or Doeg or Conoy or "Pit River" or Pomo or Kashaya or Ponca or Ponka or Pottawatomi or Bodewadmik or Powhatan or Puyallup or Spuyalepabs or Quapaw or Ugahxpa or Quechan or Yuma or Kwtsaan or Quileute or Salinan or Saponi or Monacan or Sapon or "Eastern Blackfoot" or Christanna or Sawawatodo or Serrano or Taaqtam or "Maarenga' yam" or Yuhaviatam or Shasta or Chasta or Sasti or Shoshone or Siletz or Sinkine or Sinkyone or "Siuslaw Umpqua" or Skitswish or "Schitsu' umash" or Snohomish or Snuqualmi or Sokoki or Missiquoi or Stillaguamish or Stoluckwamish or Suquamish or Sutaio or Swinomish or Skagit or Syilx or Okanagan or Sotaae or "Taga Ticutta" or Takelma or Dagelma or Taltushtuntede or Galice or "Tanan Gwich' in" or Taos or Taovaya or Tataviam or Alliklik or Tawakoni or Tahuacano or Tenino or Thawikila or Hathawekela or "Fort Ancient" or Tigua or Tillamook or Nehalem or Timbisha or Panamint or Timpanogos or Tlingit or "Toi Ticutta" or Tolowa or "Talawa Dini' " or Tongva or Gabrieleno or Fernandeno or Tobikhar or Tonkawa or Ticanwatic or Tsikip or Appalousa or Opelousa or Tsitsistas or Tubatulabal or Tukabatchee or Tuscarora or Tomahittan or Kuskarawock or Tutelo or Tutero or Totteroy or Tutera or Yusan or Tututni or Umatilla or Umpqua or Waccamaw or Waxmaw or Wadatika or "Harney Valley Paiute" or Wailiki or Waluulapam or "Walla Walla" or Walpapi or Huipui or Wampanoag or Massasoit or Wanapum or Wappo or Washoe or Wichita or Willapa or Kwalhioqua or "Wi pukba" or "Verde Valley Yavapai" or Wintu or "Northern Wintun" or Wiyot or "Wee' at" or Weyet or Yakama or "Yamosopo Tuviwarai" or Yaqui or Yoeme or Yatasi or Yattasih or "Yavbe' " or "Yavapai" or "Ysleta del Sur" or Yojuane or Yokuts or Mariposa or Yuki or Yupighyt or Yup'ik or Yupik or Yurok or "Olekwo'l" or Zuni) | 8,081 |
| S4 | ( maori or "tangata whenua" ) OR ( "new zealand" and (aborig* or indigen*) ) | 4,305 |
| S3 | ( MH "Australia" or (australia* or "northern terrritory" or tasmania or "new south wales" or victoria or queensland) ) AND ( (aborigin* or indigen*) ) | 9,625 |
| S2 | TI ( ( (MH "Eskimos") OR (MH "Native Americans") OR (MH "Indigenous Peoples+") or (MH "Health Services, Indigenous") or (MH "Indigenous Health") or (MH “Ethnopharmacology”) or Athapaskan or Saulteaux or Wakashan or Cree or Dene or Inuit or Inuk or Inuvialuit* or Haida or Ktunaxa or Tsimshian or Gitsxan or “Nisga'a” or Haisla or Heiltsuk or Oweenkeno or “Kwakwaka'wakw” or “Nuu chah nulth” or “Tsilhqot'in” or Dakelh or “Wet'suwet'en” or Sekani or “Dunne-za” or Dene or Tahltan or Kaska or Tagish or Tutchone or Nuxalk or Salish or “Stl'atlimc” or “Nlaka'pamux” or Okanagan or “Sec wepmc” or Tlingit or Anishinaabe or Blackfoot or Nakoda or Tasttine or “Tsuu T'inia” or “Gwich'in” or Han or Tagish or Tutchone or Algonquin or Nipissing or Ojibwa or Potawatomi or Innu or Maliseet or “Mi'kmaq” or Micmac or “Mic mac” or Passamaquoddy or Haudenosaunee or Cayuga or Mohawk or Oneida or Onodaga or Seneca or Tuscarora or Wyandot or Aboriginal* or Indigenous* or Metis or “red road” or "on reserve" or “off reserve” or “First Nation” or “First Nations” or Amerindian or (urban N3 (Indian* or Native* or Aboriginal*)) or ethnomedicine or “country food*” or “residential school*” or ((MH “Medicine, Traditional”) or "traditional medicine*" ) not Chinese ) or ( MH“Shamanism”) or shaman* or “traditional heal*” or “traditional food*” or “medicine man” or “medicine woman” or autochtone* or (Native* N1 (American* or man or men or women or woman or boy* or girl* or adolescent* or youth or youths or person* or adult or people* or Indian* or Nation or tribe* or tribal or band or bands)) ) ) AND ( ( (MH “Canada+”) or (Canad* or “British Columbia” or “Columbie Britannique” or Alberta or Saskatchewan or Manitoba or Ontario or Quebec or “Nova Scotia” or “New Brunswick” or Newfoundland or Labrador or “Prince Edward Island” or “Yukon Territory” or NWT or “Northwest Territories” or Nunavut or Nunavik or Nunatsiavut or NunatuKavut)) or AB ( ( (MH "Eskimos") OR (MH "Native Americans") OR (MH "Indigenous Peoples+") or (MH "Health Services, Indigenous") or (MH "Indigenous Health") or (MH “Ethnopharmacology”) or Athapaskan or Saulteaux or Wakashan or Cree or Dene or Inuit or Inuk or Inuvialuit* or Haida or Ktunaxa or Tsimshian or Gitsxan or “Nisga'a” or Haisla or Heiltsuk or Oweenkeno or “Kwakwaka'wakw” or “Nuu chah nulth” or “Tsilhqot'in” or Dakelh or “Wet'suwet'en” or Sekani or “Dunne-za” or Dene or Tahltan or Kaska or Tagish or Tutchone or Nuxalk or Salish or “Stl'atlimc” or “Nlaka'pamux” or Okanagan or “Sec wepmc” or Tlingit or Anishinaabe or Blackfoot or Nakoda or Tasttine or “Tsuu T'inia” or “Gwich'in” or Han or Tagish or Tutchone or Algonquin or Nipissing or Ojibwa or Potawatomi or Innu or Maliseet or “Mi'kmaq” or Micmac or “Mic mac” or Passamaquoddy or Haudenosaunee or Cayuga or Mohawk or Oneida or Onodaga or Seneca or Tuscarora or Wyandot or Aboriginal* or Indigenous* or Metis or “red road” or "on reserve" or “off reserve” or “First Nation” or “First Nations” or Amerindian or (urban N3 (Indian* or Native* or Aboriginal*)) or ethnomedicine or “country food*” or “residential school*” or ((MH “Medicine, Traditional”) or "traditional medicine*" ) not Chinese ) or ( MH“Shamanism”) or shaman* or “traditional heal*” or “traditional food*” or “medicine man” or “medicine woman” or autochtone* or (Native* N1 (American* or man or men or women or woman or boy* or girl* or adolescent* or youth or youths or person* or adult or people* or Indian* or Nation or tribe* or tribal or band or bands)) ) ) AND ( ( (MH “Canada+”) or (Canad* or “British Columbia” or “Columbie Britannique” or Alberta or Saskatchewan or Manitoba or Ontario or Quebec or “Nova Scotia” or “New Brunswick” or Newfoundland or Labrador or “Prince Edward Island” or “Yukon Territory” or NWT or “Northwest Territories” or Nunavut or Nunavik or Nunatsiavut or NunatuKavut)) | 3,794 |
| S1 | (MH "Indigenous Peoples+") OR (MH "Aboriginal Canadians+") OR (MH "First Nations of Canada") OR (MH "Arctic Peoples+") OR (MH "Inuit") OR (MH "First Nations of Australia+") OR (MH "Aboriginal Australians") OR (MH "Torres Strait Islanders") OR (MH "Maori") OR (MH "Native Americans+") OR (MH "Alaska Natives") | 22,957 |
