# Supplementary material for: Understanding virtual primary healthcare with Indigenous populations: a rapid evidence review
Source: BMC Health Serv Res. 2023 Mar 29;23:303. doi: 10.1186/s12913-023-09299-6 (PMC10054202; doi:10.1186/s12913-023-09299-6)
Supplement: Supplementary file 3 — Supplementary Material 3 [file 12913_2023_9299_MOESM3_ESM.docx]

**Data extraction tool**

**General questions:**

1. Name of reviewer:
2. Reference/citation:
3. Describe the Indigenous population or community identified in the source:
4. Briefly describe the program/service/intervention in the source (include the name of program/service/intervention):
5. Briefly summarize key topics covered in this source (focus on priorities)

**Document characteristics**

| 1. **Description of study sample and/or participants**    1. Sample size or number of participants (if appropriate)    2. Inclusion/exclusion criteria:    3. Age range:    4. Gender and/or sex:    5. Place of residence:  - Rural - Remote - Urban - Mix - Treaty or reserve land  1. **Study design and methods**    1. Year of publication:    2. Years of data collection (if applicable):    3. Methodological approach (e.g., qualitative, quantitative, mixed, Indigenous research methods/approaches)    4. List the outcomes reported and specific **cultural safety, PHC quality, and/or virtual PHC quality** indicators reported within the outcomes    5. Data collection methods:    6. Data analysis methods: 2. **Description of virtual care program/service/intervention:** 3. Virtual care program/service/intervention modality (e.g., phone, web-based, text messaging): 4. What are program/service/intervention aim(s): 5. Who developed and implemented the program/service/intervention? 6. Describe the delivery of the virtual care program/service/intervention (e.g., teleconference set up in health centres). 7. What type of health condition is the program/service/intervention targeting (diabetes, mental health, oncology etc)? 8. Indigenous focused or Indigenous-led?    1. 🞎 Focused    2. 🞎 Led 9. Are any relevant contextual details provided or described by the authors (e.g., historical factors, colonization, racism, discrimination)? 10. Was there mention of sustainability (plans for long-term success) or capacity building (enabling communities or Indigenous communities to be sustainable)? If yes describe. 11. **Extracting key findings** 12. Study outcomes: 13. Qualitative themes or concepts identified by authors within the study: 14. Authors’ interpretations of their data in discussion: 15. Author-identified limitations: 16. Virtual care models/frameworks developed (e.g., were there any models that were developed as a result of the findings) 17. Facilitators/enablers to success of the virtual care program/service/intervention that were identified by authors: 18. Identification of barriers to success of the virtual care program/service/intervention or contextual changes (e.g., policy change, change in government, an event such as a crisis or emergency, mobilization of an issue by policy entrepreneur or advocacy group demonstrated by the release of a policy report or statement) that hindered the delivery, implementation, and sustainability of the project identified by the authors 19. Identification of contextual or environmental enablers that supported the implementation of the virtual care program/service/intervention (e.g., supportive policy environment, community readiness, federal/provincial/state relationship, allocation of resources, workforce supply, etc.) 20. **High-level summary of key messages/insights from the source: [Please include and label your interpretation of the source]**     1. Did the program do what the authors expected it would? (i.e., did it work?)   🞎 Yes  🞎 No   - 1. Author summary:   2. Reviewer summary: |
| --- |
